# Network Meta-Analysis of Perioperative Analgesic Effects of Different Interventions on Postoperative Pain After Arthroscopic Shoulder Surgery Based on Randomized Controlled Trials

**DOI:** 10.3389/fmed.2022.921016

**Published:** 2022-07-08

**Authors:** Wu Jiangping, Quan Xiaolin, Shu Han, Xiaolan Zhou, Nie Mao, Deng Zhibo, Gong Ting, Hu Shidong, Li Xiangwei, Yuan Xin, Shu Guoyin

**Affiliations:** ^1^Center for Joint Surgery, Department of Orthopedic Surgery, The Second Affiliated Hospital of Chongqing Medical University, Chongqing, China; ^2^Chongqing Clinical Research Center for Geriatrics and Gerontology, Chongqing, China; ^3^Medical Record Statistics Section, The Second Hospital of Chongqing Medical University, Chongqing, China

**Keywords:** arthroscopic shoulder surgery, postoperative pain, network meta-analysis, randomized controlled trials, pair-wise meta-analysis

## Abstract

**Background:**

Shoulder arthroscopic surgery is a common surgical method used in orthopedics. However, severe postoperative pain can significantly limit the early joint movement of patients and adversely affect the impact of the surgery. At present, there is no consistent and effective analgesic scheme for the management of postoperative pain after arthroscopic surgery of the shoulder.

**Purpose:**

The aim of this study was to search for the most effective analgesic scheme to control pain in the perioperative period of arthroscopic surgery of the shoulder.

**Study Design:**

Network meta-analysis.

**Methods:**

We searched 5 different databases (i.e., Medline, PubMed, Embase, Web of Science, and the Cochrane Library) from January 2011 to January 2021 for English literature. Thereafter, we sifted out randomized controlled trials (RCTs), which compared different intervention schemes for pain management after shoulder arthroscopy and selected only 12 h, 24 h, or 48 h after the patient leaves the operating room as an optimal period for administration of analgesic intervention schemes. Only patients with shoulder disease who have undergone arthroscopic shoulder surgery were included in this study. The Cochrane “risk of bias” was used for the quality assessment. Moreover, some additional tests were performed to enhance the credibility of the results.

**Results:**

Twenty-nine RCTs involving 1,885 patients were included in this frequentist network meta-analysis (NMA). These articles mainly were divided into two distinct groups, namely, the nerve block group and the non-nerve block group. Regarding the nerve block group, at postoperative 12 h, the intervention suprascapular nerve block + interscalene nerve block (SSNB + INB) was ranked first, whereas INB + intra-articular injection (INB + IAI) was ranked first at 24 h and 48 h postoperation. In the non-nerve block group, external application (EA) was ranked first at postoperative 12 h, but oral administration (OA) exhibited a better analgesic effect at postoperative 24 h and postoperative 48 h.

**Conclusion:**

We conclude that the analgesic effect of SSNB+INB was the best at postoperative 12 h, and INB+IAI was the best at postoperative 24 h and 48 h in the nerve block group. For the non-nerve block group, the effect of EA was the best at postoperative 12 h, and the analgesic effect of OA at postoperative 24 h and 48 h was significantly better than any other interventions.

**Systematic Review Registration:**

https://www.crd.york.ac.uk/prospero/, identifier: CRD42021286777.

## Introduction

Shoulder pain has become a common musculoskeletal disease, in which the rotator cuff gets torn, and a frozen shoulder is commonly observed. Despite the well-documented postoperative pain, a disturbing sensory and emotional experience linked with actual or potential tissue damage can occur, which might develop within the first 48 h (1). Shoulder arthroscopic surgery is one of the most frequently performed surgeries in orthopedics with multitudinous surgical indications, such as rotator cuff tears, instability, and frozen shoulder (2–4). Postoperative pain can significantly limit the early activity of patients, thereby affecting the clinical effect of the operation. Thus, effective pain management after arthroscopic shoulder surgery can allow patients to get discharged earlier, reduce the risk for readmission, and thereby improve the ultimate outcome after surgery (5, 6). Currently, two main measures, i.e., subjective pain scales and quantity of postoperative narcotic consumption, are used to assess the patient pain levels. At present, the pain scales used in the mainstream include the visual analog scale (VAS) and numeric rating scale (NRS), which are both repeatable and reliable, depending on the subjective patient reporting (7, 8).

A number of previous studies have evaluated different kinds of available postoperative pain management strategies after arthroscopic surgery of the shoulder (3, 5, 9, 10). These include oral administration (OA), intra-articular injection (IAI), external application (EA), intravenous administration (IVA), and regional nerve block, which can yield different analgesic conclusions. For instance, Toma et al. (10) recommended that interscalene brachial plexus blockade could be the first-choice regional analgesic technique. Michell Ruiz-Suarez and Barber (5) reported that postoperative pain management should include three distinct stages, namely, preoperative, intraoperative, and postoperative.

Moreover, preemptive analgesia with oral medications can be taken before operation (11), a regional nerve block can be used during operation (12), and an analgesic pump can be used after operation (13). At present, two kinds of analgesia, namely, single analgesia and multimode analgesia are mainly used; however, which analgesic scheme among these two has the best effect remains unclear.

Some traditional systematic reviews have focused on this topic, but they have only included two therapies or did not effectively compare the analgesic efficacy of a combination of the numerous intervention measures due to limitations in the methodology used (3, 14–16). In addition, there are also some meta-analyses that have been examined on this topic. Changjiao et al. (16) and Kay et al. (17) have reported that the analgesic effect of interscalene nerve block (INB) was significantly better than suprascapular nerve block (SSNB), and SSNB can be an alternative to INB. Ul Huda et al. (15) suggested that preoperative use of gabapentin might effectively reduce the incidence of postoperative nausea and vomiting, whereas White et al. (14) reported that anterior SSNB could display fewer complications than INB. The latter also suggested that anterior SSNB could be more suitable for shoulder arthroscopic surgery in terms of complications. However, there was no accepted and consistent conclusion reached based on all these prior studies. This study aimed to explore the most effective analgesic scheme that can be employed in the perioperative period of shoulder arthroscopy through network meta-analysis (NMA).

## Methods

This NMA was performed according to the PRISMA (Preferred Reporting Items for Systematic Reviews and Meta-Analyses) guidelines (18), and our review was registered with PROSPERO (CRD42021286777).

### Eligibility Criteria

We included RCTs of patients with shoulder disease for comparing the different interventions used in pain management after shoulder arthroscopy. The selected intervention types included the following: eight regional nerve blocks, IAI, IVA, OA, and EA. Regional nerve block included INB, SSNB, axillary nerve block (ANB), supraclavicular nerve block (SCNB), stellate ganglion block (SGB), infraclavicular-suprascapular block (ICSCB), and costoclavicular block (CCB). In addition to the analgesic methods of high thoracic erector spine plane block (HTESPB), CEBs have been found to be similar to regional nerve block methods, and therefore, they were also classified as the regional nerve block group. IAIs of narcotic drugs, such as bupivacaine, magnesium sulfate, and liposomal bupivacaine, were also considered. It has been established that the subacromial injection anesthetics can communicate with the joint during surgery; therefore, we also attributed subacromial injection to IAIs, such as Merivirta et al. (19, 20). IVA included intravenous ketoprofen and intravenous ketamine, oral drugs included oral ibuprofen or pregabalin, EA included fentanyl patch, and some interventions were a combination of the above. Refer to Table 1 for the intervention groups in detail, and we have classified all the interventions into two distinct types, including the nerve block group with nerve block during the surgery and the non-nerve block group without nerve block in one surgery.

**Table 1:** Interventions on postoperative pain after arthroscopic shoulder surgery studied in this network meta-analysis.

	**Nerve block group**	**Non-nerve block group**
Interventions	SSNB+ANB	IAI
	INB+SSNB	OA
	INB+OA	IVA
	INB+IAI	EA
	INB	-
	CEB	
	SGB	
	CCB	
	ANB	
	SCNB	
	SSNB	
	HTESPB	
	ICSCB	

*INB, interscalene nerve block; SCNB, supraclavicular nerve block; SSNB, suprascapular nerve block; HTESPB, high thoracic erector spinae plane block; CEB, cervical epidural block; SGB, stellate ganglion block; ICSCB, infraclavicular-suprascapular blocks; CCB, costoclavicular blocks; ANB, axillary nerve block; IAI, intra-articular injection; OA, oral administration; EA, external application; IVA, intravenous administration*.

The inclusion criteria consisted of the following:

#### Patient

Those who have been diagnosed with shoulder joint diseases, such as rotator cuff tears, instability, and frozen shoulder, and underwent shoulder arthroscopic surgery, regardless of age, sex, course of the disease, underlying diseases, and other differences among the various groups in the same study.

#### Experimental Design

It consisted of the comparison of the two intervention measures (Table 1).

#### Outcome Measures

The determination of VAS or the NRS at postoperative 12 h, 24 h, and 48 h.

#### Study Design

RCTs that have reported different intervention measures in the management of postoperative pain.

### Systematic Search

We extensively searched English articles in Medline, PubMed, Embase, Web of Science, and the Cochrane library using the following keywords: arthroscopic shoulder surgery, postoperative pain, pain, therapeutics, and randomized controlled trial (RCT). The search was carried out by using the combination of the keywords above and their free words, and all databases were set from January 2011 to January 2021.

### Study Selection

We (W.J.P. and D.Z.B.) assessed the credibility of these potential articles with the above criteria and resolved the differences after consulting and discussing with the senior author (N.M.). Finally, useful data were extracted independently and reviewed by the senior author.

### Data Extraction

The extracted data included publication time, author, article and intervention type, the characteristics of the subjects, mean patient age, the ratio of the male to female, the number of patients in each arm, male percentage, outcome representation method, and time point of outcome index. The outcome index selected by us was the value of postoperative pain score, which was divided into three distinct groups, namely, postoperative 12 h, postoperative 24 h, and postoperative 48 h, according to the time point of the outcome, and the outcome was expressed as mean ± standard difference (M ± SD). Both VAS and NRS were scored 0–10, so it was deemed appropriate to include them in the same meta-analysis (15).

### Quality Assessment

The Cochrane “risk of bias” tool was used to evaluate the methodological quality of the selected articles (21).

### Statistical Analysis

Pooling the different instruments that report on a common domain typically is conducted by converting each instrument to SD units and combining their effects across the studies as the standardized mean difference (SMD). However, this approach has major limitations, including difficulties in interpretation and vulnerability to the baseline heterogeneity of enrolled patients (22, 23). Therefore, by using the linear transformation and assuming that instruments reporting on the shared domains might have similar measurement properties, we converted all the measures of pain intensity and physical functioning to 10-cm VASs (24), such as Rothe et al. (25).

Initially, we performed a conventional pairwise meta-analysis by using a DerSimonian–Laird random-effects model and then conducted a frequentist NMA by using the methodology of the multivariate meta-analysis by assuming a common heterogeneity parameter (26), using the mv-meta command and the network suite in Stata (SE 15.1) (27). The results were expressed by mean difference (MD) and 95% CI.

In addition, the ranking probabilities for all the different protocols were calculated, and the results were reported as the (surface under the cumulative ranking curve) (28): 100% meant the best treatment, whereas 0% meant the worst treatment. We ranked the analgesic effects of the various intervention measures, after combining them with the outcomes of the NMA.

### Inconsistency Analysis

We calculated the inconsistency between the direct and indirect evidence at home and abroad by evaluating the potential differences in all the closed loops of the network and by comparing the suitability and conciseness of consistency and inconsistency of the models (27), which was assessed using the node-splitting method (29).

### Additional Analysis

Publication bias was analyzed by using Egger's test. We screened the studies with a sample size of <40 patients in order to conduct the sensitivity analyses and calculated the rank probabilities again. The results were considered reliable in case of the insignificant difference between the latter and the former outcomes. A comparison-adjusted funnel plot was then plotted to evaluate the risk of bias as an asymmetric plot can only indicate a small study effect (28).

## Results

### Eligible Studies

**Figure 1:**
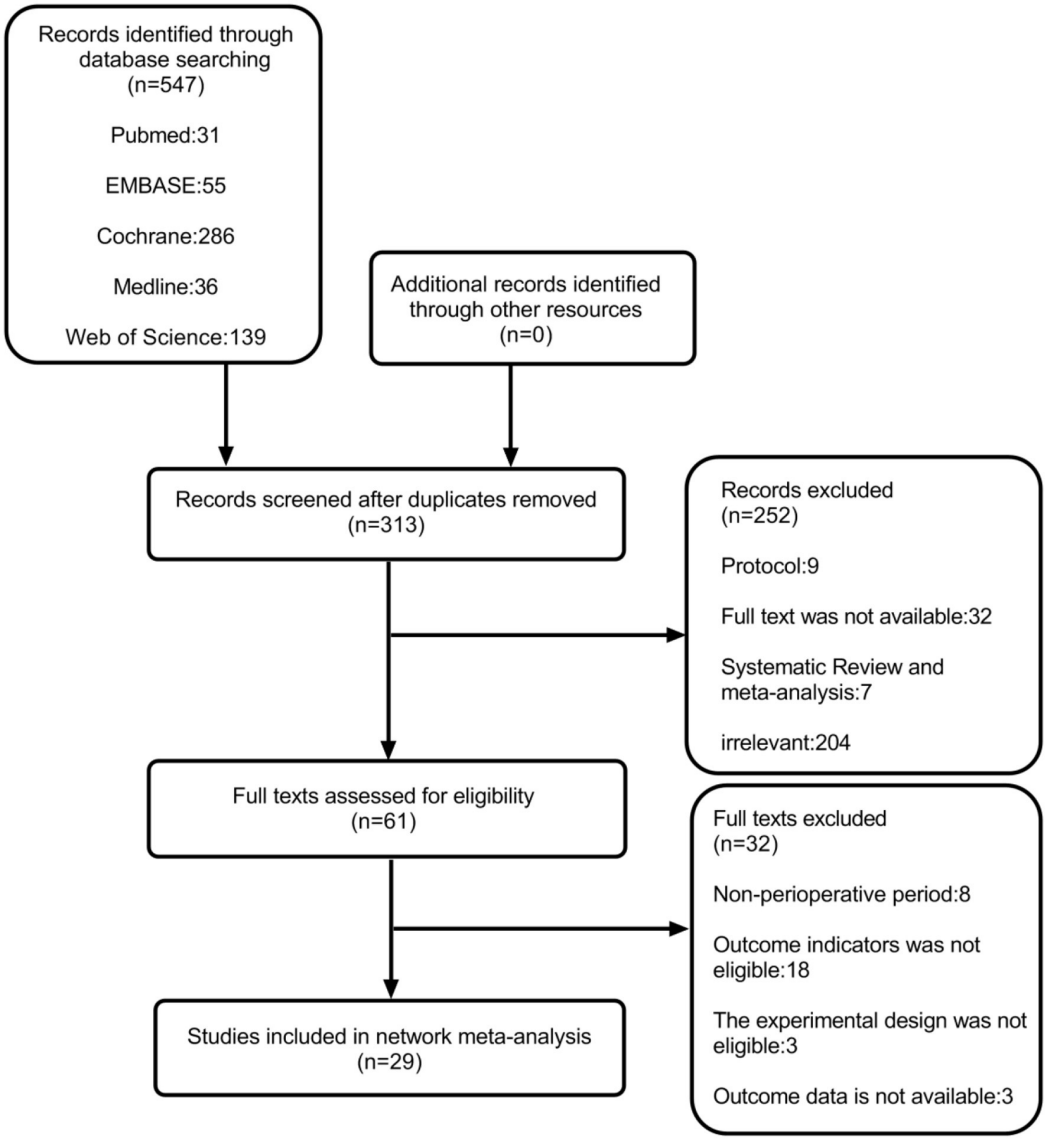
PRISMA flowchart of study selection.

**Figure 2:**
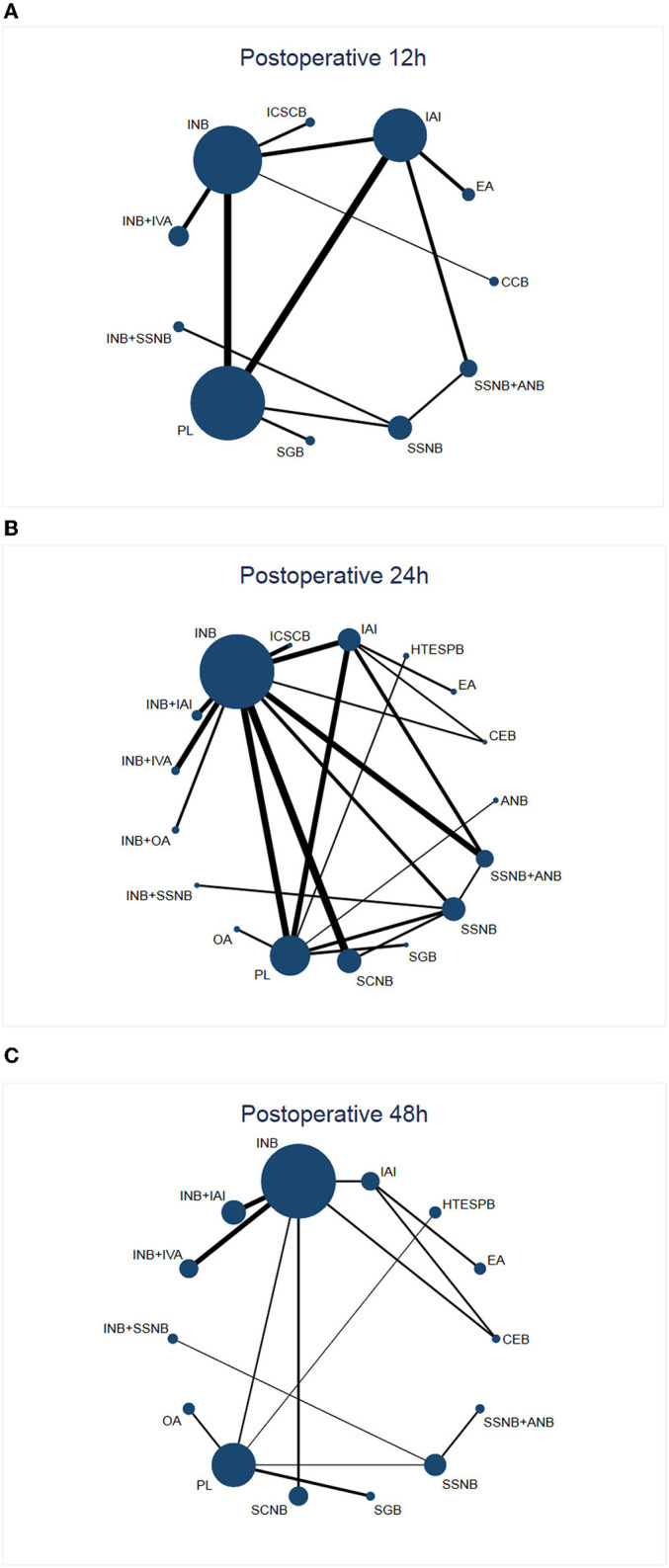
Network plot of treatment comparisons. **(A)** (Network 1) Network plot of treatment comparisons for postoperative 12 h. The size of the blue area indicates the sample size of each group, and the thickness indicates the results of comparisons between two groups. **(B)** (Network 2) Network plot of treatment comparisons for postoperative 24 h. The size of the blue area indicates the sample size of each group, and the thickness indicates the results of comparisons between two groups. **(C)** (Network 3) Network plot of treatment comparisons for postoperative 48 h. The size of the blue area indicates the sample size of each group, and the thickness indicates the results of comparisons between two groups. INB, interscalene nerve block; SSNB, suprascapular nerve block; SCNB, supraclavicular nerve block; ICSCB, infraclavicular-suprascapular blocks; CCB, costoclavicular blocks; ANB, axillary nerve block; IAI, intra-articular injection; EA, external application; IVA, intravenous administration; SGB, stellate ganglion block; PL, placebo; HTESPB, high thoracic erector spinae plane block; CEB, cervical epidural block; ANB, axillary nerve block; OA, oral administration.

After a systematic search, 547 records were found, among which we included only 29 reports that were published between 2011 and 2021 (Figure 1) (11, 12, 19, 20, 25, 30–53). Among these 29 articles, the average number of patients per article was 65 (range, 30–114), and the average age varied from 29 to 63 years. Generally speaking, we included 1,885 patients, and in Table 2, we have summarized the key details of each article. Of these 29 articles, 12 articles used the NRS 0–10 score scale, 16 articles used the VAS 0–10 score scale, and 1 article used NRS 0–100 score scale (25). The results of 15 articles described the pain scores at postoperative 12 h, 28 articles included scores at postoperative 24 h, and 14 articles included scores at 48 h after the surgery (Figure 2). The network for eligible comparisons of the three different groups is presented in Figure 2.

**Table 2:** Characteristics of the included studies.

**Study**	**Design**	**Patients**	**Sample size (T1/T2 or T1/T2/T3)**	**Age (years, T1/T2 or T1/T2/T3, M ± SD)**	**Gender (T1/T2 or T1/T2/T3; M/F)**	**Intervention**	**Pain score**	**Outcome time point (post-operative time)**
Sowoon et al. (11)	RCT	Arthroscopic shoulder surgery (Bankart or rotator cuff repair)	30/30	55 ± 9/51 ± 12	(13/17)/(13/17)	OA/PL	NRS	24h; 48h
Auyong et al. (33)	RCT	Unilateral shoulder arthroscopic surgery (rotator cuff or Bankart repair)	63/63/63	54 ± 13/53 ± 14/55 ± 14	(38/25)/(39/24)/(42/21)	INB/SCNB	NRS	24h
Bahadir et al. (36)	RCT	Unilateral arthroscopic shoulder surgery	30/30	47.6 ± 13.01/49 ± 10.26	(10/20)/(16/14)	HTESPB/PL	VAS	24h; 48h
Lee et al. (43)	RCT	Arthroscopic rotator cuff repair (rotator cuff tear)	24/24	57.4 ± 9.6 /57.3 ± 12.0	(12/12)/(8/16)	INB+SSNB/SSNB	VAS	12h; 24h; 48h
Lee et al. (12)	RCT	Arthroscopic rotator cuff repair (rotator cuff tear)	15/15	48.9 ± 11.7/51.6 ± 10.6	(11/4)/(10/5)	SSNB/PL	VAS	12h; 24h
Merivirta et al. (19)	RCT	Arthroscopic surgery (reparable rotator cuff tear)	30/30	52 ± 9/54 ± 9	(11/19)/(14/16)	EA/IAI	NRS	12h; 24h; 48h
Merivirta et al. (20)	RCT	Arthroscopic surgery (subacromial impingement disease)	39/43	53 ± 9/55 ± 6	(24/15)/(34/9)	IAI/PL	NRS	12h; 24h
Anneleen et al. (45)	RCT	Elective arthroscopic shoulder surgery	50/48	54 ± 10/51 ± 10	(28/22)/(18/30)	INB/SSNB+ANB	NRS	24h
Park et al. (47)	RCT	Arthroscopic shoulder operations	19/19/19	52 ± 13/53 ± 9/54 ± 7	not mentioned	INB/IAI	NRS	24h; 48h
Sethi et al. (49)	RCT	Arthroscopic rotator cuff repair surgery	25/25	not mentioned	not mentioned	INB+IAI/INB	VAS	24h; 48h
Thompson et al. (50)	RCT	Arthroscopic Bankart repair	40/40	29.9 ± 10.1/32.6 ± 10.8	(27/13)/(25/15)	INB+OA/INB	VAS	24h
Verdecchia (51)	RCT	Arthroscopic rotator cuff repair	42/42	58.2 ± 7.2/56.2 ± 7.8	(15/27)/(15/27)	INB+IAI/INB	NRS	24h; 48h
Woo (53)	RCT	Arthroscopic shoulder operations	20/20	42.85 ± 18.97/49.65 ± 14.11	(15/5)/(12/8)	INB+IVA/INB	NRS	12h; 24h; 48h
Aksu et al. (30)	RCT	Arthroscopic shoulder surgery	20/20/20	45.1 ± 15.5/44.2 ± 15.9/43.4 ± 13.5	(13/7)/(12/8)/(13/7)	INB/IAI	VAS	12h; 24h
Choi et al. (35)	RCT	Arthroscopic rotator cuff repair	20/20	47.3 ± 13.3/49.1 ± 11.1	(11/9)/(10/10)	SGB/PL	VAS	12h; 24h; 48h
Jeske et al. (40)	RCT	Arthroscopic subacromial decompression	15/15	59.1 ± 6.1/63.6 ± 9.0	(9/6)/(8/7)	SSNB/PL	VAS	24h; 48h
Lee et al. (42)	RCT	Arthroscopic rotator cuff repairs(rotator cuff tears)	21/21	54.0 ± 8.0/55.8 ± 8.0	(14/7)/(14/7)	SSNB+ANB/SSNB	VAS	12h; 24h; 48h
Liu et al. (44)	RCT	Arthroscopic rotator cuff repair(rotator cuff tear)	31/31	59.74 ± 5.85/56.77 ± 7.29	(17/14)/(15/16)	INB/PL	VAS	12h; 24h; 48h
Derya OZKAN (2020)	RCT	Arthroscopic shoulder surgery	22/21	58.5 ± 7.9/53.7 ± 16.5	(7/15)/(10/11)	SSNB+ANB/IAI	NRS	12h; 24h
Tuba Berra Saritas et al. (48)	RCT	Arthroscopic rotator cuff repair	30/30	39.8 ± 9.2/41.6 ± 10.4	(17/13)/(14/16)	IAI/PL	VAS	12h; 24h
Julian Aliste et al. (32)	RCT	Arthroscopic shoulder surgery	20/20	50.6 ± 8.0/57.9 ± 9.3	(11/9)/(9/11)	INB/ICSCB	NRS	12h; 24h
Aliste (32)	RCT	Arthroscopic shoulder surgery	20/20	54.72 ± 12.1/53.5 ± 10.4	(10/12)/(8/14)	INB/CCB	VAS	12h
Cabaton et al. (34)	RCT	Arthroscopic rotator cuff repair	52/51	57.67 ± 10.67/59 ± 8.38	(32/20)/(27/24)	SCNB/INB	NRS	24h; 48h
Dhir et al. (38)	RCT	Arthroscopic shoulder surgery	30/29	51.3 ± 14.2/46.5 ± 14.5	(26/4)/(22/7)	INB/SSNB+ANB	NRS	24h
Gurger et al. (39)	RCT	Arthroscopic rotator cuff repair	43/42	58.47 ± 7.18/58.21 ± 7.67	(18/25)/(20/22)	PL/INB	VAS	12h; 24h
Kumara et al. (41)	RCT	Arthroscopic shoulder surgeries	30/30	not mentioned	not mentioned	INB/SSNB	VAS	24h
Rothe et al. (25)	RCT	Arthroscopic subacrimial decompression	27/23	53 ± 10.5/54 ± 10.5	(11/16)/(11/12)	ANB/PL	VAS	24h
Wiesmann et al. (52)	RCT	Arthroscopic shoulder surgery	56/58	53.0 ± 13/52.7 ± 13	(34/22)/(34/24)	SCNB/INB	NRS	24h
Demir (37)	RCT	Arthroscopic shoulder surgery	30/30	48.03 ± 11.79/46.73 ± 12.50	(14/16)/(18/12)	INB/INB+IVA	VAS	12h; 24h; 48h

*INB, interscalene nerve block; SCNB, supraclavicular nerve block; SSNB, suprascapular nerve block; HTESPB, high thoracic erector spinae plane block; CEB, cervical epidural block; SGB, stellate ganglion block; ICSCB, infraclavicular-suprascapular blocks; CCB, costoclavicular blocks; ANB, axillary nerve block; IAI, intra-articular injection; OA, oral administration; EA, external application; IVA, intravenous administration; VAS, visual analog scale; NRS, numeric rating scale; M ± SD, mean ± standard deviation; RCT, randomized controlled trial; PL, placebo*.

### Quality Assessment

**Figure 3:**
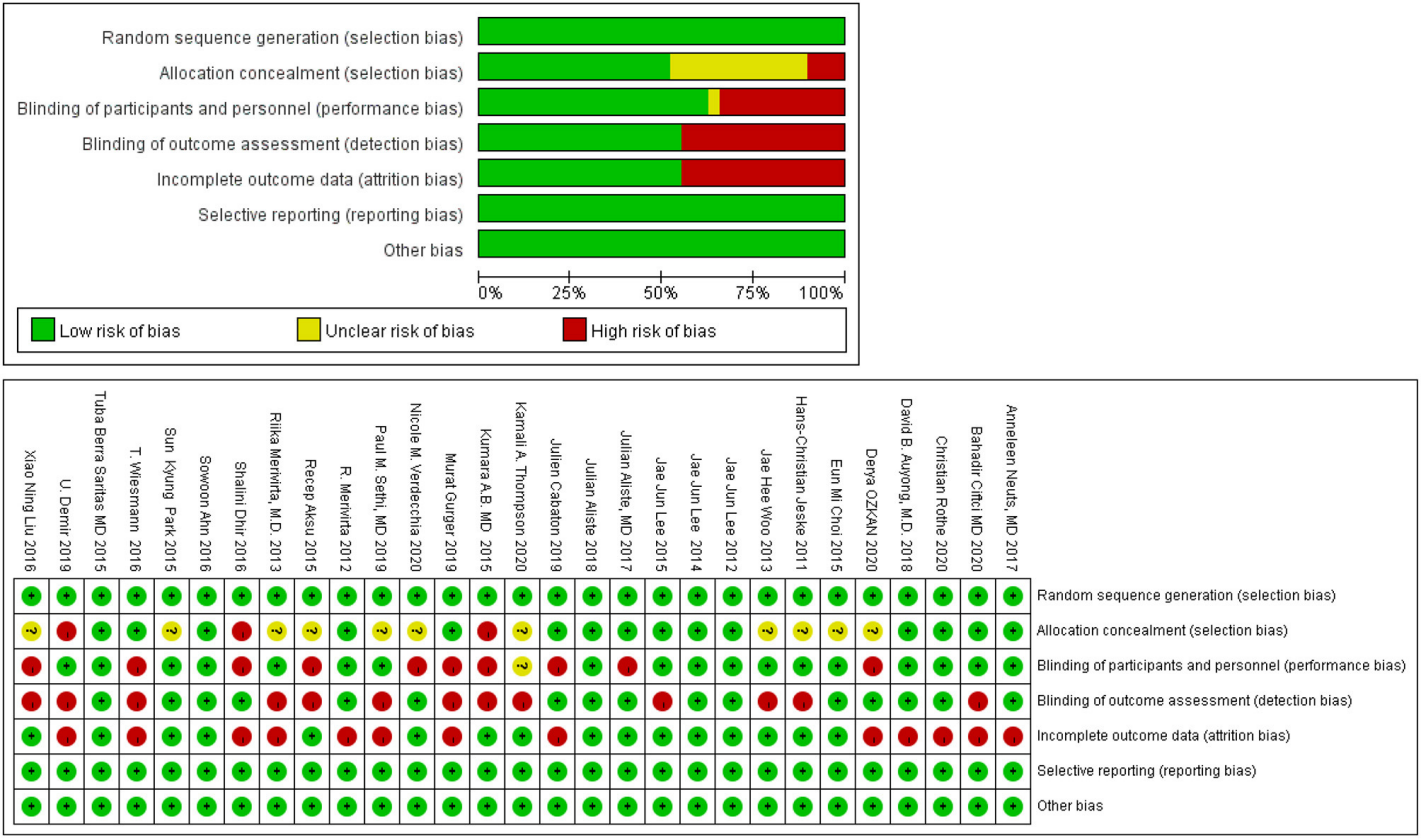
Quality assessment.

**Figure 4:**
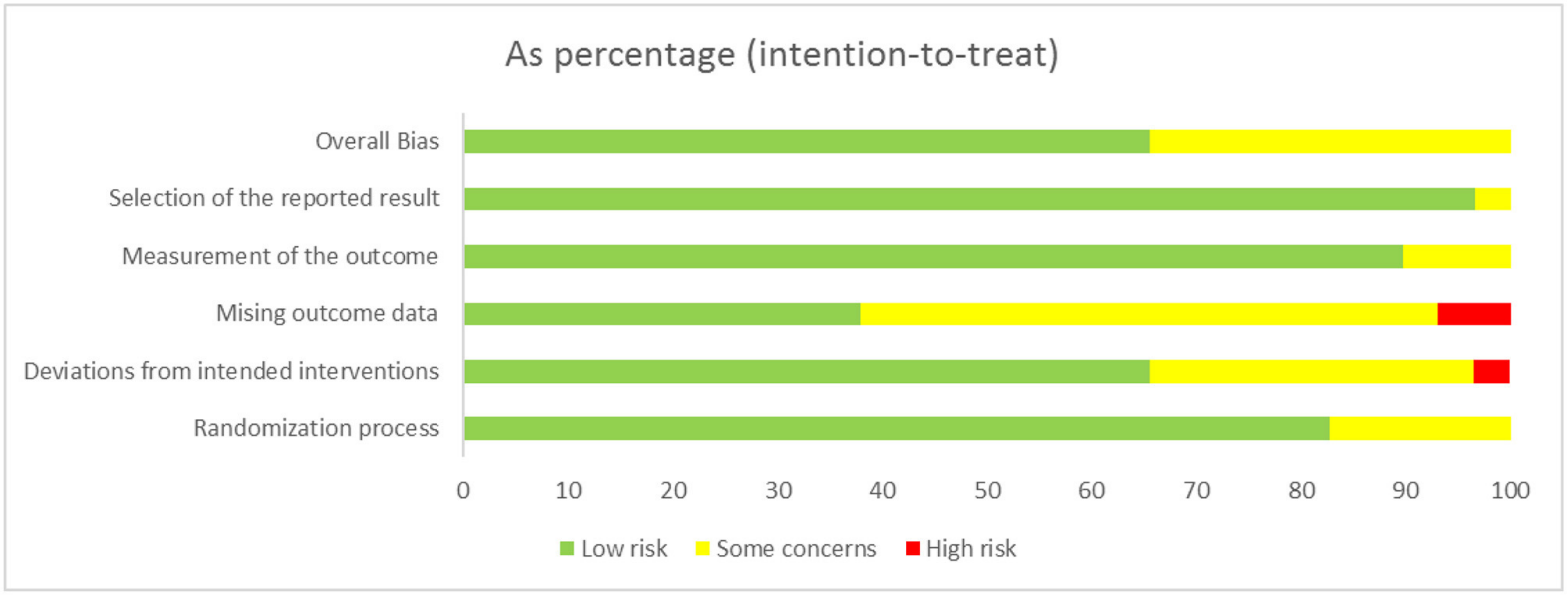
Quality assessment (ROB2.0).

We found that no study was highly risky for the random sequence generation and selective reporting after being assessed for the quality. A total of 52% were considered to have a low risk for allocation concealment, whereas 45% of the studies had a high risk for incomplete results, and none of the studies displayed a high risk for selective reporting. A total of 62% of the literature implemented blind methods for experimenters and subjects, 55% of the recorders were blind, and among them, 38% of the articles applied the blind method for all the participants. The detailed results are shown in Figure 3. We also used the ROB2.0 risk assessment tool to assess the quality of incorporated references, the detailed results are shown in Figure 4 and Table 3. Finally, we used GRADE criteria to assess the quality of evidence (Table 4).

**Table 3:** Quality assessment (ROB2.0).

**Intention–to–treat**	**Unique ID**	**Study ID**	**Experimental**	**Comparator**	**Outcome**	**Weight**	**D1**	**D2**	**D3**	**D4**	**D5**	**Overall**
	Sowoon et al. (11)	NA	NA	NA	NA	1	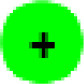	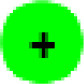	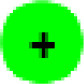	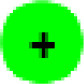	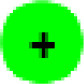	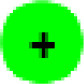
	Auyong et al. (33)	NA	NA	NA	NA	1	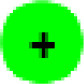	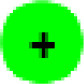	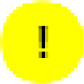	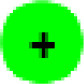	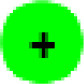	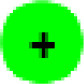
	Bahadir et al. (36)	NA	NA	NA	NA	1	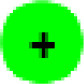	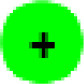	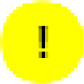	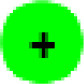	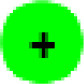	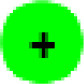
	Lee et al. (43)	NA	NA	NA	NA	1	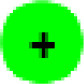	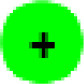	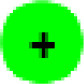	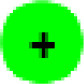	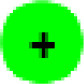	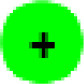
	Lee et al. (12)	NA	NA	NA	NA	1	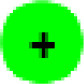	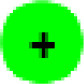	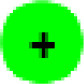	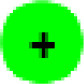	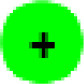	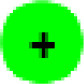
	Merivirta et al. (19)	NA	NA	NA	NA	1	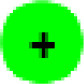	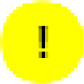	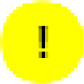	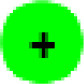	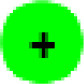	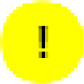
	Merivirta et al. (20)	NA	NA	NA	NA	1	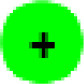	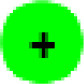	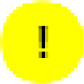	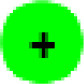	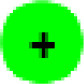	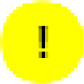
	Anneleen et al. (45)	NA	NA	NA	NA	1	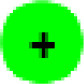	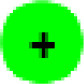	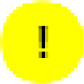	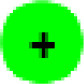	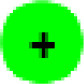	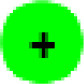
	Park et al. (47)	NA	NA	NA	NA	1	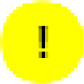	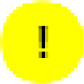	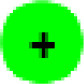	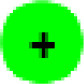	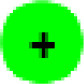	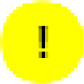
	Sethi et al. (49)	NA	NA	NA	NA	1	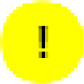	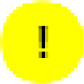	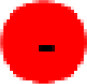	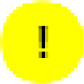	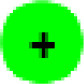	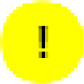
	Thompson et al. (50)	NA	NA	NA	NA	1	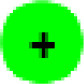	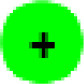	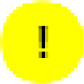	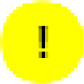	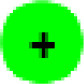	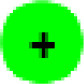
	Verdecchia (51)	NA	NA	NA	NA	1	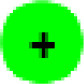	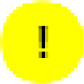	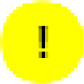	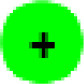	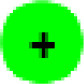	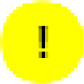
	Woo (53)	NA	NA	NA	NA	1	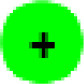	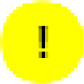	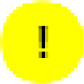	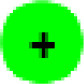	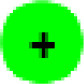	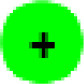
	Aksu et al. (30)	NA	NA	NA	NA	1	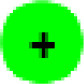	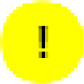	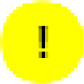	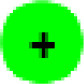	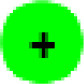	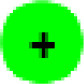
	Choi et al. (35)	NA	NA	NA	NA	1	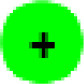	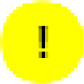	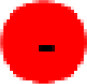	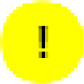	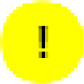	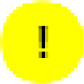
	Jeske et al. (40)	NA	NA	NA	NA	1	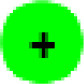	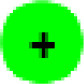	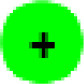	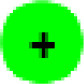	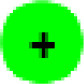	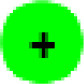
	Lee et al. (42)	NA	NA	NA	NA	1	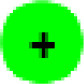	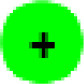	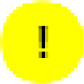	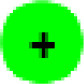	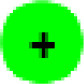	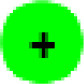
	Liu et al. (44)	NA	NA	NA	NA	1	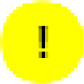	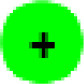	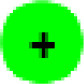	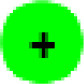	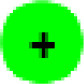	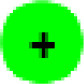
	Özkan et al. (46)	NA	NA	NA	NA	1	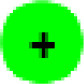	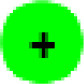	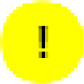	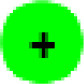	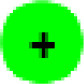	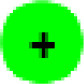
	Tuba Berra Saritas et al. (48)	NA	NA	NA	NA	1	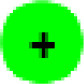	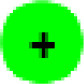	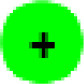	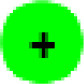	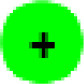	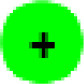
	Julian Aliste et al. (32)	NA	NA	NA	NA	1	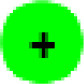	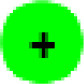	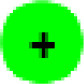	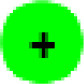	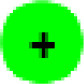	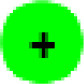
	Aliste (32)	NA	NA	NA	NA	1	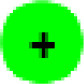	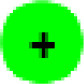	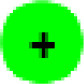	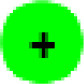	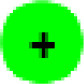	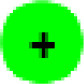
	Cabaton et al. (34)	NA	NA	NA	NA	1	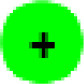	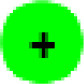	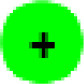	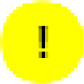	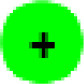	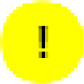
	Dhir et al. (38)	NA	NA	NA	NA	1	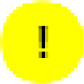	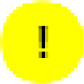	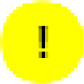	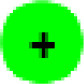	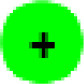	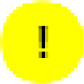
	Gurger et al. (39)	NA	NA	NA	NA	1	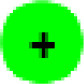	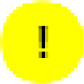	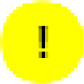	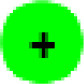	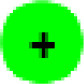	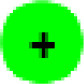
	Kumara et al. (41)	NA	NA	NA	NA	1	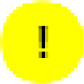	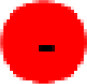	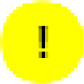	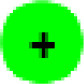	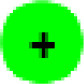	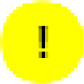
	Rothe et al. (25)	NA	NA	NA	NA	1	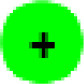	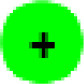	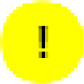	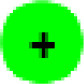	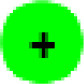	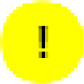
	Wiesmann et al. (52)	NA	NA	NA	NA	1	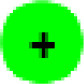	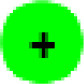	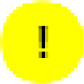	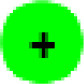	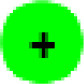	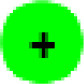
	Demir (37)	NA	NA	NA	NA	1	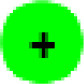	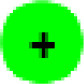	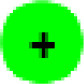	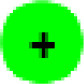	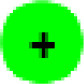	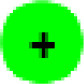

*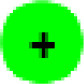 Low risk, 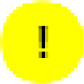 Some concerns, 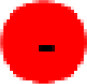 High risk, D1 Randomisation process, D2 Randomisation process, D3 Missing outcome data, D4 Measurement of the outcome, D5 Selection of the reported result*.

**Table 4:** Quality of evidence according to the GRADE criteria.

		**Characteristics of the included studies**					
**Outcomes**	**No. of studies**	**Risk of bias**	**Inconsistency^**a**^**	**Indirectness**	**Imprecision**	**Publication bias**	**Overall GRADE quality score**
VAS at postoperative 12h	15	Not serious	Not serious	Not serious	serious	None	⊕⊕⊕O Moderate
VAS at postoperative 24h	28	Not serious	Not serious	Not serious	Not serious	None	⊕⊕⊕ Advanced
VAS at postoperative 48h	14	Serious	Not serious	Not serious	Not serious	None	⊕⊕⊕O Moderate

*VAS, Visual Analog Scale Score. ^a^Indicates studies differed in the age of participants and in the detailed postoperative interventions*.

### Pair-Wise Meta-Analysis

We entered all the data that were suitable for the traditional pairwise meta-analysis into STATA 15.1, developed random-effects models, and then evaluated the SMDs and 95% CIs.

All the data, which were suitable for the conventional pairwise meta-analysis, were entered into STATA 15.1, and then the random-effects models were developed. Thereafter, the SMDs and 95% CIs were evaluated. In the postoperative 12 h group, 17 pairs of pain score comparisons were performed among which 9 had 95% CIs beyond the null value, thus suggesting significant differences, as follows: 1 pair of INB + SSNB vs. SSNB (SMD −1.34, 95% CI −1.97 to −0.71), 3 pairs of IAI vs. placebo (PL) (SMD −0.39, 95% CI −0.71 to −0.08), 3 pairs of INB vs. PL (SMD −0.94, 95% CI −1.49 to −0.39), 1 pair of SSNB+ANB vs. SSNB (SMD −0.81, 95% CI −1.44 to −0.18), and 1 pair of SSNB+ANB vs. IAI (SMD −0.79, 95% CI −1.41 to −0.17). The differences in the remaining 8 comparisons were considered insignificant. Regarding the postoperative 24 h group, 34 pairs of pain score comparisons were performed while 9 of which had 95% CIs beyond the null value, thus suggesting significant differences, as follows: 1 pair of OA vs. PL (SMD −1.49, 95% CI −2.06 to −0.91), 1 pair of HTESPB vs.PL (SMD −0.80, 95% CI −1.33 to −0.27), 1 pair of INB + SSNB vs. SSNB (SMD −0.62, 95% CI 0.40 to 2.44), 3 pairs of IAI vs.PL (SMD −0.40, 95% CI −0.71 to −0.09), 1 pair of CEB to IAI (SMD −2.23, 95% CI −3.36 to −1.09), 1 pair of INB vs. CEB (SMD 1.42,95% CI 0.40 to 2.44), and 1 pair of ANB vs. PL (SMD −1.17, 95% CI −1.78 to −0.57). We found no significant differences in the remaining 25 comparisons. Regarding the postoperative 48 h group, 16 pairs of pain score comparisons were performed among which 7 had 95% CIs beyond the null value, thus suggesting significant differences, as follows: 1 pair of OA vs. PL (SMD −0.94, 95% CI −1.47 to −0.40), 1 pair of HTESPB vs. PL (SMD −0.80, 95% CI −1.32 to −0.27), 1 pair of INB + SSNB vs. SSNB (SMD −0.93, 95% CI −1.53 to −0.33), 1 pair of INB vs. IAI (SMD −1.88, 95% CI −2.98 to −0.78), 1 pair of CEB vs. IAI (SMD −2.27, 95% CI −3.45 to −1.09), and 2 pairs of INB + IAI vs. INB (SMD −0.64, 95% CI −0.98 to −0.29). We found no significant differences in the remaining 9 comparisons. We have shown the results in the upper triangle of Tables 5–7, and the significant differences have been shaded.

**Table 5:** Results of treatment comparisons for postoperative 24 h.

A				*N =* 1;0.00 (−0.62; 0.62)						
0.45 (0.04;5.05)	B	*N =* 1;0.05 (−0.46; 0.56)								
0.52 (0.11;2.51)	1.16 (0.18;7.33)	C		*N =* 1;0.00 (−0.88; 0.88)			*N =* 3;−0.39 (−0.71; −0.08)			*N =* 1;−0.79 (−1.41; −0.17)
1.29 (0.18;9.26)	2.87 (0.20;40.92)	2.47 (0.36;16.78)	D	*N =* 1;0.13 (−0.49; 0.75)						
1.00 (0.31;3.20)	2.24 (0.27;18.73)	1.92 (0.67;5.56)	0.78 (0.16;3.84)	E	*N =* 2;−0.08 (−1.20; 1.05)		*N =* 3;−0.94 (−1.49; −0.39)			
1.23 (0.27;5.68)	2.74 (0.26;28.49)	2.36 (0.56;10.00)	0.96 (0.15;6.26)	1.23 (0.45;3.32)	F					
5.42 (0.51;58.14)	12.09 (0.81;181.01)	10.41 (1.43;75.58)	4.21 (0.31;57.43)	5.41 (0.68;42.83)	4.41 (0.45;43.54)	G			*N =* 1;−1.34(−1.97;−0.71)	
0.30 (0.08;1.23)	0.68 (0.09;5.01)	0.58 (0.27;1.27)	0.24 (0.04;1.40)	0.30 (0.14;0.66)	0.25 (0.07;0.87)	0.06 (0.01;0.38)	H	*N =* 1;0.58 (−0.06; 1.21)	*N =* 1;−0.21 (−0.92; 0.51)	
0.11 (0.01;0.87)	0.24 (0.02;3.00)	0.21 (0.04;1.16)	0.08 (0.01;0.88)	0.11 (0.02;0.61)	0.09 (0.01;0.64)	0.02 (0.00;0.23)	0.36 (0.08;1.65)	I		
0.54 (0.08;3.60)	1.21 (0.12;12.04)	1.04 (0.27;4.11)	0.42 (0.05;3.75)	0.54 (0.12;2.41)	0.44 (0.07;2.64)	0.10 (0.02;0.42)	1.79 (0.50;6.40)	5.00 (0.68;36.72)	J	*N =* 1;−0.81 (−1.44; −0.18)
2.59 (0.35;19.48)	5.79 (0.56;59.49)	4.98 (1.19;20.77)	2.02 (0.20;19.98)	2.59 (0.50;13.46)	2.11 (0.31;14.32)	0.48 (0.07;3.21)	8.52 (1.98;36.69)	23.86 (2.88;197.92)	4.77 (1.36;16.69)	K

*1. Lower–left triangle presents the findings (MD with 95%CI) of the network meta–analysis conducted using Stata 15.1. Upper–right triangle presents the findings (SMD with 95% CI) of the pair–wise meta–analyses conducted using STATA 15.1 and N refers to the numbers of RCTs which compared the 2 interventions directly. 3. A positive MD favors the lower–right intervention; a negative MD favors the upper–left intervention.4. Statistically significant findings are shaded. A, ANB (axillary nerve block); B, CEB (cervical epidural block); C, EA (external application); D, HTESPB (high thoracic erector spinae plane block); E, IAI (intra–articular injection); F, ICSCB (infraclavicular–suprascapular blocks); G, INB (interscalene nerve block); H, INB+IAI (interscalene nerve block + intra–articular injection); I, INB+IVA (interscalene nerve block + intravenous administration); J, INB+OA (interscalene nerve block + oral administration); K, INB+SSNB (interscalene nerve block + suprascapular nerve block); L, OA (oral administration); M, PL (placebo); N, SCNB (supraclavicular nerve block); O= SGB (stellate ganglion block); P, SSNB (suprascapular nerve block); Q, SSNB+ANB (suprascapular nerve block + axillary nerve block).*

*Red represents the code of intervention measures and green represents the significant difference between two intervention measures with statistical significance*.

**Table 6:** Results of treatment comparisons for postoperative 24 h.

A												*N =* 1;−1.17 (−1.78; −0.57)				
2.74 (0.49;15.36)	B			*N =* 1;−2.23 (−3.36; −1.09)		*N =* 1;1.42 (0.40; 2.44)										
0.65 (0.09;4.71)	0.24 (0.04;1.58)	C		*N =* 1;−0.16 (−0.67; 0.35)												
1.06 (0.19;5.94)	0.39 (0.06;2.31)	1.62 (0.21;12.36)	D									*N =* 1;−0.80 (−1.33; −0.27)				
0.47 (0.12;1.80)	0.17 (0.05;0.58)	0.73 (0.17;3.12)	0.45 (0.11;1.85)	E		*N =* 2;−0.44 (−1.09; 0.20)						*N =* 3;−0.40 (−0.71; −0.09)				*N =* 1;−0.15 (−0.75; 0.45)
1.03 (0.09;11.65)	0.38 (0.04;4.04)	1.58 (0.12;21.38)	0.98 (0.08;11.56)	2.17 (0.25;18.84)	F	*N =* 1;−0.24 (−0.86; 0.38)										
0.53 (0.14;1.96)	0.19 (0.06;0.65)	0.81 (0.16;4.09)	0.50 (0.12;2.02)	1.11 (0.55;2.26)	0.51 (0.07;3.93)	G	*N =* 2;−1.05 (−2.82; 0.73)	*N =* 2;−0.43 (−1.16; 0.31)	*N =* 1;0.16 (−0.28; 0.60)			*N =* 3;−0.42 (−1.24; 0.39)	*N =* 3;0.17 (−0.07; 0.40)		*N =* 2;−0.03 (−0.38; 0.33)	*N =* 2;−0.44 (−0.98; 0.11)
3.48 (0.66;18.36)	1.27 (0.26;6.20)	5.34 (0.79;36.25)	3.30 (0.58;18.58)	7.35 (2.12;25.46)	3.38 (0.35;33.00)	6.60 (2.39;18.25)	H									
1.27 (0.23;7.03)	0.46 (0.09;2.38)	1.95 (0.28;13.79)	1.21 (0.20;7.10)	2.69 (0.73;9.89)	1.24 (0.12;12.48)	2.42 (0.81;7.18)	0.37 (0.08;1.62)	I								
0.35 (0.05;2.68)	0.13 (0.02;0.92)	0.54 (0.06;5.08)	0.33 (0.04;2.68)	0.75 (0.14;4.08)	0.34 (0.03;4.43)	0.67 (0.14;3.14)	0.10 (0.02;0.64)	0.28 (0.04;1.83)	J							
1.32 (0.19;9.20)	0.48 (0.07;3.31)	2.03 (0.23;17.94)	1.25 (0.17;9.23)	2.79 (0.55;14.12)	1.28 (0.10;16.46)	2.51 (0.54;11.63)	0.38 (0.06;2.40)	1.04 (0.16;6.83)	3.74 (0.42;32.95)	K					*N =* 1;−0.62 (−1.20; −0.04)	
5.78 (0.91;36.61)	2.11 (0.31;14.16)	8.86 (1.05;74.93)	5.47 (0.81;36.80)	12.20 (2.57;58.03)	5.61 (0.44;72.36)	10.97 (2.35;51.27)	1.66 (0.26;10.55)	4.54 (0.69;30.03)	16.36 (1.85;144.95)	4.38 (0.53;35.80)	L	*N =* 1;−1.49 (−2.06; −0.91)				
0.35 (0.11;1.14)	0.13 (0.04;0.45)	0.54 (0.11;2.64)	0.33 (0.09;1.18)	0.74 (0.39;1.40)	0.34 (0.04;2.85)	0.67 (0.37;1.21)	0.10 (0.03;0.33)	0.28 (0.08;0.96)	0.99 (0.19;5.19)	0.27 (0.06;1.25)	0.06 (0.01;0.25)	M		*N =* 1;0.11 (−0.51; 0.73)	*N =* 2;−0.13 (−0.63; 0.38)	
0.33 (0.07;1.53)	0.12 (0.03;0.52)	0.51 (0.08;3.10)	0.31 (0.06;1.56)	0.70 (0.24;2.04)	0.32 (0.04;2.91)	0.63 (0.27;1.44)	0.09 (0.03;0.35)	0.26 (0.07;1.02)	0.94 (0.16;5.40)	0.25 (0.05;1.36)	0.06 (0.01;0.32)	0.94 (0.35;2.53)	N		*N =* 1;0.30 (−0.20; 0.79)	
0.28 (0.04;2.02)	0.10 (0.01;0.78)	0.44 (0.05;4.08)	0.27 (0.04;2.03)	0.60 (0.11;3.27)	0.28 (0.02;3.88)	0.54 (0.10;2.89)	0.08 (0.01;0.58)	0.22 (0.03;1.66)	0.81 (0.08;7.88)	0.22 (0.02;1.95)	0.05 (0.01;0.41)	0.81 (0.17;3.90)	0.86 (0.13;5.51)	O		
0.49 (0.13;1.88)	0.18 (0.05;0.67)	0.75 (0.14;3.99)	0.46 (0.11;1.93)	1.03 (0.45;2.35)	0.47 (0.06;4.00)	0.92 (0.49;1.75)	0.14 (0.04;0.47)	0.38 (0.11;1.36)	1.38 (0.26;7.32)	0.37 (0.09;1.48)	0.08 (0.02;0.41)	1.38 (0.71;2.70)	1.47 (0.56;3.87)	1.71 (0.31;9.40)	P	*N =* 1;−0.52 (−1.13; 0.10)
1.12 (0.25;4.99)	0.41 (0.10;1.70)	1.72 (0.30;9.89)	1.06 (0.22;5.09)	2.37 (0.91;6.21)	1.09 (0.12;9.85)	2.13 (0.93;4.87)	0.32 (0.09;1.20)	0.88 (0.22;3.48)	3.18 (0.55;18.31)	0.85 (0.16;4.42)	0.19 (0.04;1.06)	3.20 (1.27;8.02)	3.40 (1.08;10.67)	3.94 (0.64;24.31)	2.31 (0.96;5.57)	Q

*1. Lower–left triangle presents the findings (MD with 95%CI) of the network meta–analysis conducted using Stata 15.1. Upper–right triangle presents the findings (SMD with 95% CI) of the pair–wise meta–analyses conducted using STATA 15.1 and N refers to the numbers of RCTs which compared the 2 interventions directly. 3. A positive MD favors the lower–right intervention; a negative MD favors the upper–left intervention. 4. Statistically significant findings are shaded. A, CEB (cervical epidural block); B, EA (external application); C, HTESPB (high thoracic erector spinae plane block); D, IAI (intra–articular injection); E, INB (interscalene nerve block); F, INB+IAI (interscalene nerve block + intra–articular injection); G, INB+IVA (interscalene nerve block + intravenous administration); H, INB+SSNB (interscalene nerve block + suprascapular nerve block); I, OA (oral administration); J, PL (placebo); K, SCNB (supraclavicular nerve block); L, SGB (stellate ganglion block); M, SSNB (suprascapular nerve block); N, SSNB+ANB (suprascapular nerve block + axillary nerve block).*

*Red represents the code of intervention measures and green represents the significant difference between two intervention measures with statistical significance*.

**Table 7:** Results of treatment comparisons for postoperative 48 h.

A		*N =* 1,−2.27 (−3.45;−1.09)	*N =* 1,0.38 (−0.55;1.31)									
0.12 (0.02,0.77)	B		*N =* 1,0.00 (−0.51;0.51)										
0.80 (0.10,6.17)	6.51 (0.57,74.22)	C							*N =* 1,−0.80 (−1.32; −0.27)				
0.12 (0.03,0.44)	1.00 (0.27,3.77)	0.15 (0.02,1.18)	D	*N =* 1,−1.88 (−2.98; −0.78)									
0.71 (0.19,2.57)	5.76 (0.91,36.43)	0.88 (0.18,4.34)	5.76 (1.60,20.74)	E	*N =* 2,−0.64 (−0.98; −0.29)	*N =* 2,−0.53 (−1.58;0.53)			*N =* 1–0.34 (−0.84;0.16)	*N =* 1,−0.34 (−0.84;0.16)			
2.73 (0.54,13.80)	22.28 (2.76,179.75)	3.42 (0.53,22.14)	22.29 (4.45,111.72)	3.87 (1.45,10.29)	F								
1.81 (0.34,9.70)	14.72 (1.74,124.72)	2.26 (0.33,15.48)	14.73 (2.76,78.59)	2.56 (0.87,7.52)	0.66 (0.15,2.83)	G							
0.80 (0.08,8.00)	6.51 (0.46,92.69)	1.00 (0.16,6.17)	6.52 (0.65,64.99)	1.13 (0.17,7.65)	0.29 (0.03,2.50)	0.44 (0.05,3.99)	H					*N =* 1,−0.93 (−1.53; −0.33)	
1.96 (0.22,17.14)	16.01 (1.27,202.40)	2.46 (0.48,12.67)	16.01 (1.84,139.19)	2.78 (0.49,15.89)	0.72 (0.10,5.31)	1.09 (0.14,8.47)	2.46 (0.35,17.34)	I	*N =* 1,−0.94 (−1.47; −0.40)				
0.44 (0.08,2.55)	3.57 (0.40,32.22)	0.55 (0.19,1.56)	3.57 (0.62,20.64)	0.62 (0.19,2.06)	0.16 (0.03,0.75)	0.24 (0.05,1.22)	0.55 (0.12,2.43)	0.22 (0.06,0.79)	J		*N =* 1,0.03 (−0.59;0.65)	*N =* 1,0.18 (−0.54;0.90)	
1.29 (0.19,8.54)	10.50 (1.05,105.29)	1.61 (0.20,13.27)	10.50 (1.59,69.19)	1.82 (0.46,7.27)	0.47 (0.09,2.57)	0.71 (0.12,4.12)	1.61 (0.15,17.07)	0.66 (0.07,6.07)	2.94 (0.47,18.35)	K			
0.42 (0.04,4.32)	3.40 (0.23,49.82)	0.52 (0.08,3.35)	3.40 (0.33,35.09)	0.59 (0.08,4.16)	0.15 (0.02,1.36)	0.23 (0.02,2.15)	0.52 (0.06,4.45)	0.21 (0.03,1.56)	0.95 (0.20,4.44)	0.32 (0.03,3.55)	L		
0.40 (0.05,3.07)	3.23 (0.28,36.93)	0.50 (0.11,2.18)	3.24 (0.42,24.93)	0.56 (0.11,2.76)	0.15 (0.02,0.94)	0.22 (0.03,1.51)	0.50 (0.17,1.43)	0.20 (0.04,1.04)	0.91 (0.32,2.58)	0.31 (0.04,2.54)	0.95 (0.15,6.13)	M	*N =* 1,−0.38 (−0.99;0.23)
0.65 (0.06,7.19)	5.33 (0.35,82.37)	0.82 (0.12,5.68)	5.33 (0.49,58.46)	0.93 (0.12,7.01)	0.24 (0.03,2.27)	0.36 (0.04,3.60)	0.82 (0.16,4.22)	0.33 (0.04,2.62)	1.49 (0.29,7.63)	0.51 (0.04,5.90)	1.57 (0.17,14.80)	1.65 (0.47,5.76)	N

*1. Lower-left triangle presents the findings (MD with 95%CI) of the network meta-analysis conducted using Stata 15.1. Upper-right triangle presents the findings (SMD with 95% CI) of the pair-wise meta-analyses conducted using STATA 15.1 and N refers to the numbers of RCTs which compared the 2 interventions directly. 3. A positive MD favors the lower-right intervention; a negative MD favors the upper-left intervention. 4. Statistically significant findings are shaded. A, CEB (cervical epidural block); B, EA (external application); C, HTESPB (high thoracic erector spinae plane block); D, IAI (intra-articular injection); E, INB (interscalene nerve block); F, INB+IAI (interscalene nerve block + intra-articular injection); G, INB+IVA (interscalene nerve block + intravenous administration); H, INB+SSNB (interscalene nerve block + suprascapular nerve block); I, OA (oral administration); J, PL (placebo); K, SCNB (supraclavicular nerve block); L, SGB (stellate ganglion block); M, SSNB (suprascapular nerve block); N, SSNB+ANB (suprascapular nerve block + axillary nerve block).*

*Red represents the code of intervention measures and green represents the significant difference between two intervention measures with statistical significance*.

### Network Meta–Analysis

All the differences of the possible comparisons were evaluated, and the results as the MDs and 95% CIs were obtained, which have been listed in the lower triangle of Tables 5–7 with the various significant differences being shaded.

Regarding the postoperative 12 h group, among the significant results, INB + SSNB vs. SSNB, INB vs. PL, SSNB + ANB vs. SSNB, and SSNB + ANB vs. IAI exhibited similar results to those of the above traditional meta–analysis. However, 1 comparison–AI vs. PL–had no significant difference, which is the difference between the NMA and the traditional meta–analysis, in which the difference between different interventions vs. PL was compared, and it was found that the top two interventions were SSNB + ANB (MD 0.06, 95% CI 0.01 to 0.38) and INB + SSNB (MD 0.12, 95% CI 0.03 to 0.51).

Regarding the postoperative 24 h group, among the significant results, OA vs. PL, CEB vs. IAI, and INB vs. CEB exhibited similar results to those of the above–discussed traditional meta–analysis. In addition, 4 distinct comparisons that included HTESPB vs. PL, INB + SSNB vs. SSNB, IAI vs. PL, and ANB vs. PL exhibited no significant differences, which may be due to the variation between the NMA and the traditional meta–analysis. Among these results, the differences between the various interventions vs. PL were also compared, and it was found that the top two interventions were OA (MD 0.06, 95% CI 0.01 to 0.25) and INB + IAI (MD 0.10, 95% CI 0.03 to 0.33).

Regarding the postoperative 48 h group, among the observed significant results, OA vs. PL, INB vs. IAI, CEB vs. IAI, and INB+IAI vs. INB exhibited similar results to those of the above–reported traditional meta–analysis. Moreover, 2 comparisons that consisted of HTESPB vs. PL and INB + SSNB vs. SSNB exhibited no significant differences, which might be due to the variation between the NMA and the traditional meta–analysis. We adapted the above steps and found that the top two interventions were INB + IAI (MD 0.16, 95% CI 0.03 to 0.75) and OA (MD 0.22, 95% CI 0.06 to 0.79).

### Rank Probability

**Figure 5:**
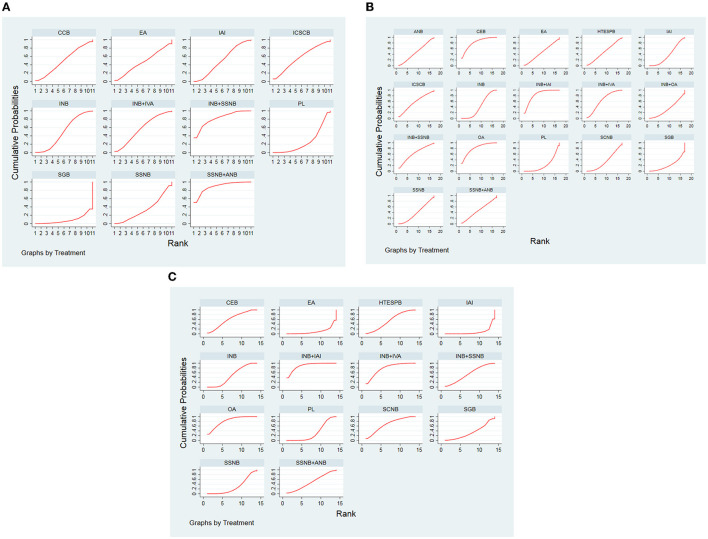
**(A)** SUCRA for results of postoperative 12 h. **(B)** SUCRA for results of postoperative 24 h. **(C)** SUCRA for results of postoperative 48 h. The area under the curve represents the cumulative rank probability of each treatment; with larger areas signifying higher probabilities. INB, interscalene nerve block; SCNB, supraclavicular nerve block; SSNB, suprascapular nerve block; HTESPB, high thoracic erector spinae plane block; CEB, cervical epidural block; SGB, stellate ganglion block; ICSCB, infraclavicular-suprascapular blocks; CCB, costoclavicular blocks; ANB, axillary nerve block; IAI, intra-articular injection; OA, oral administration; EA, external application; IVA, intravenous administration; PL, placebo.

The order of the curative effect of the intervention measures was obtained after the calculation. Based on the area under the curve, we could find out about intervention, which was the most effective (Figure 5).

For the postoperative 12 h group, the best analgesic effect was found in SSNB + ANB, whereas in the non–nerve block group, EA was ranked first.

Regarding the postoperative 24 h and 48 h group, the analgesic effect of INB + IAI was best among other treatment options in the nerve block, but OA ranked first at postoperative 12 h.

### Inconsistency Analyses

**Figure 6:**
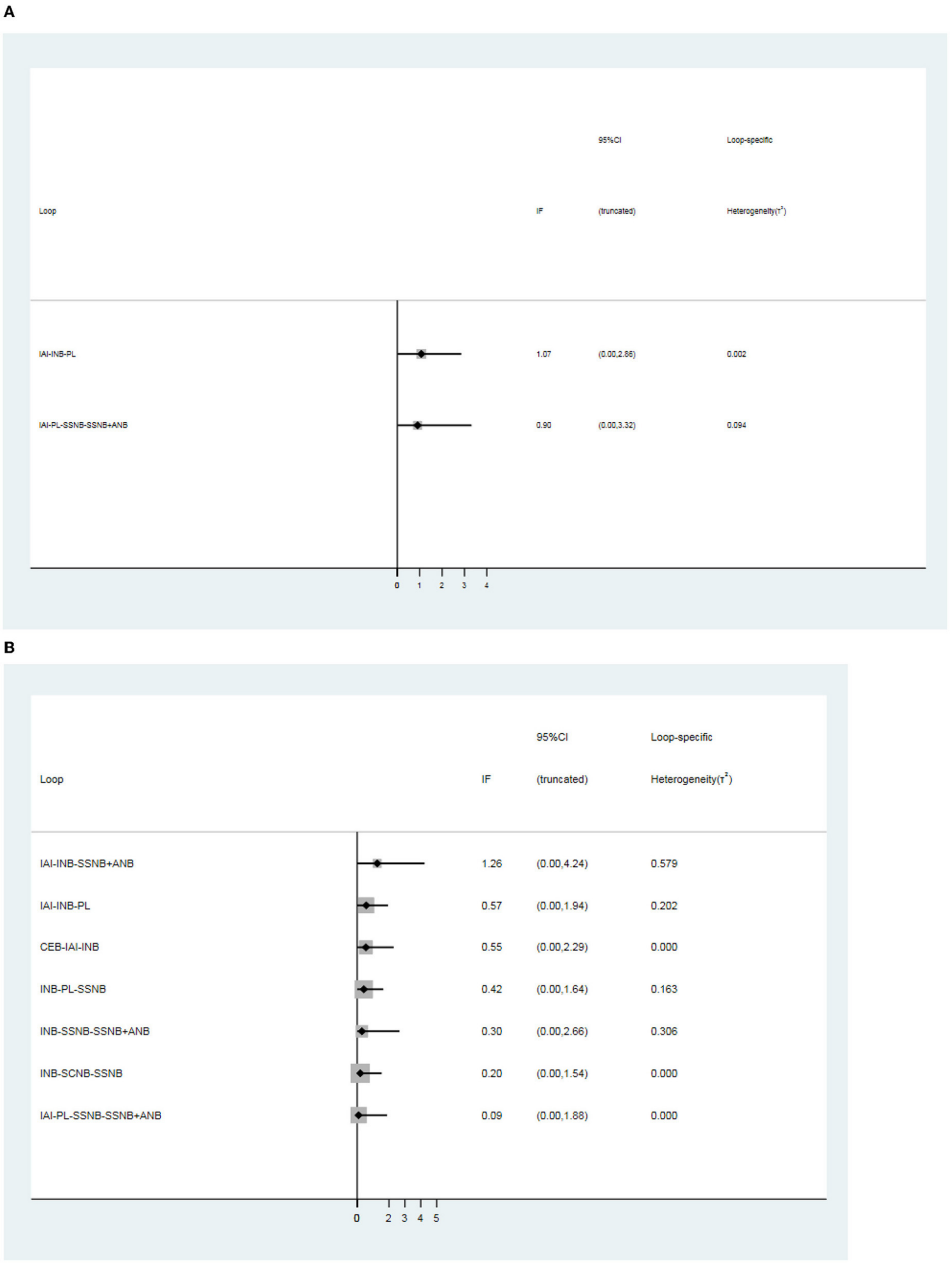
Loops analysis for inconsistency of network meta-analysis. [**(A)** postoperative 12 h., **(B)** postoperative 24 h]. When the 95% confidence interval (CI) includes 0; it means that inconsistency is low risk. INB, interscalene nerve block; SCNB, supraclavicular nerve block; SSNB, suprascapular nerve block; CEB, cervical epidural block; ANB, axillary nerve block; IAI, intra-articular injection; PL, placebo.

There was 1 quadrilateral loop (IAI–PL–SSNB–SSNB + ANB) and 1 triangle loop (IAI–INB–PL) in network 1. In network 2, 1 quadrilateral loop (IAI–PL–SSNB–SSNB + ANB) and 6 different triangle loops (IAI–INB–PL, IAI–INB–SSNB + ANB, CEB–IAI–INB, INB–PL–SSNB, INB–SSNB–SSNB + ANB, and INB–SCNB–SSNB) were found. In network 3, 1 triangle loop (CEB–IAI–INB) was found, but the triangle loop (CEB–IAI–INB) was disregarded, which was derived from the same article, and testing inconsistency in network 3 was not needed. The evaluation of inconsistency of network 1 and network 2 at the global showed no significant inconsistency, with *p*–values of 0.86 and 0.99, respectively. There was no consistency observed in these loops of network 1 and network 2 (Figure 6). In addition, no inconsistency was found between any comparison pairs in network 1 and network 2 through the node–splitting test (Tables 8, 9).

**Table 8:** Node–splitting test for inconsistency of network meta–analysis (postoperative 12 h).

**Side**	**Direct**		**Indirect**		**Difference**		**P>|z|**
	**Coef**.	**Std. Err**.	**Coef**.	**Std. Err**.	**Coef**.	**Std. Err**.	
A E*	0.00	0.59	−0.65	10.73	0.65	10.75	0.95
B C*	−0.15	0.94	1.30	185.01	−1.45	185.01	0.99
C E	0.01	1.03	−0.89	0.63	0.90	1.21	0.46
C H	0.60	0.46	0.24	1.23	0.36	1.31	0.78
C K	−2.05	0.98	−0.97	1.19	−1.08	1.54	0.48
D E*	0.25	0.81	0.00	120.49	0.25	120.50	1.00
E F*	−0.21	0.51	−0.03	71.53	−0.17	71.53	1.00
E H*	1.25	0.41	−0.17	1.94	1.42	1.98	0.47
G J*	2.30	0.73	1.22	107.55	1.08	107.56	0.99
H I*	1.03	0.78	−2.38	104.35	3.41	104.35	0.97
H J	−0.30	0.79	−1.38	1.32	1.08	1.54	0.48
J K	−1.30	0.77	−2.38	1.34	1.08	1.54	0.48

*A, CCB (costoclavicular blocks); B, EA (external application); C, IAI (intra–articular injection); D, ICSCB (infraclavicular–suprascapular blocks); E, INB (interscalene nerve block); F, INB+IVA (interscalene nerve block + intravenous administration); G, INB+SSNB (interscalene nerve block + suprascapular nerve block); H, PL (placebo); I, SGB (stellate ganglion block) J, SSNB (suprascapular nerve block); K, SSNB+ANB (suprascapular nerve block + axillary nerve block)*.

*All the evidence about these contrasts comes from the trials which directly compare them.

**Table 9:** Node–splitting test for inconsistency of network meta–analysis (postoperative 24 h).

**Side**	**Direct**		**Indirect**		**Difference**		**P>|z|**
	**Coef**.	**Std. Err**.	**Coef**.	**Std. Err**.	**Coef**.	**Std. Err**.	
A M*	1.05	0.60	0.40	8.08	0.65	8.10	0.94
B E*	2.07	0.68	0.30	1.48	1.77	1.63	0.28
B G*	1.32	0.69	3.09	1.47	−1.77	1.63	0.28
C E*	0.32	0.74	1.50	123.05	−1.18	123.05	0.99
D M*	1.10	0.65	2.09	84.13	−0.99	84.13	0.99
E G*	−0.51	0.54	0.24	0.50	−0.75	0.74	0.31
E M	0.44	0.38	−0.13	0.67	0.57	0.77	0.46
E Q	−0.38	0.96	−1.05	0.59	0.67	1.13	0.55
F G*	0.67	1.04	1.28	176.14	−0.61	176.14	1.00
G H*	−1.89	0.52	−1.24	84.76	−0.65	84.76	0.99
G I*	−0.88	0.56	−1.27	88.58	0.38	88.58	1.00
G J*	0.40	0.79	−1.28	161.37	1.68	161.37	0.99
G M	0.47	0.42	0.31	0.49	0.15	0.65	0.81
G N*	0.45	0.46	0.69	1.51	−0.24	1.56	0.88
G P	−0.03	0.48	0.18	0.49	−0.21	0.69	0.76
G Q	−1.04	0.59	−0.43	0.65	−0.61	0.88	0.49
K P*	1.00	0.71	1.44	101.21	−0.44	101.21	1.00
L M*	2.80	0.73	2.09	117.02	0.71	117.02	1.00
M O*	0.21	0.80	−2.09	118.29	2.30	118.29	0.98
M P	−0.10	0.47	−0.61	0.53	0.51	0.71	0.47
N P	−0.59	0.76	−0.23	0.68	−0.35	1.02	0.73
P Q	−0.70	0.71	−0.95	0.62	0.25	0.94	0.79

*A, ANB (axillary nerve block); B, CEB (cervical epidural block); C, EA (external application); D, HTESPB (high thoracic erector spinae plane block); E, IAI (intra–articular injection); F, ICSCB (infraclavicular–suprascapular blocks); G, INB (interscalene nerve block); H, INB+IAI (interscalene nerve block + intra–articular injection); I, INB+IVA (interscalene nerve block + intravenous administration); J, INB+OA (interscalene nerve block + oral administration); K, INB+SSNB (interscalene nerve block + suprascapular nerve block); L, OA (oral administration); M, PL (placebo); N, SCNB (supraclavicular nerve block); O= SGB (stellate ganglion block); P, SSNB (suprascapular nerve block); Q, SSNB+ANB (suprascapular nerve block + axillary nerve block)*.

*All the evidence about these contrasts comes from the trials which directly compare them.

### Additional Analysis

**Figure 7:**
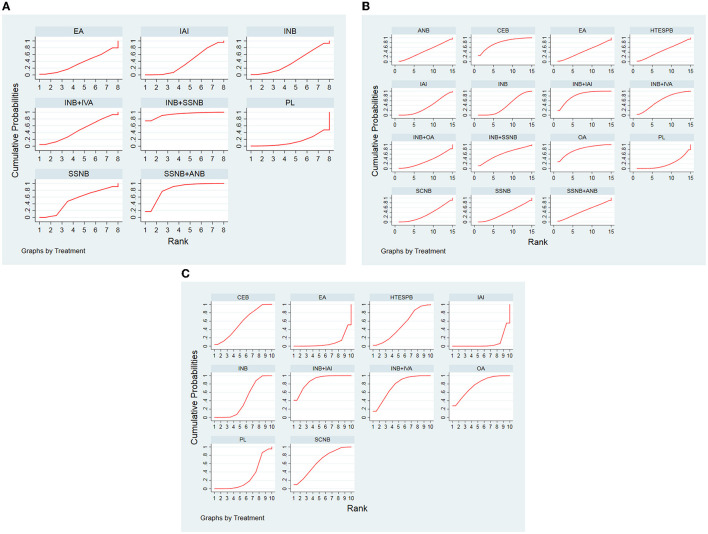
**(A)** SUCRA for results of postoperative 12 h. **(B)** SUCRA for results of postoperative 24 h. **(C)** SUCRA for results of postoperative 48 h. (*After removing the article with less than 40 people*). The area under the curve represents the cumulative rank probability of each treatment; with larger areas signifying higher probabilities. INB, interscalene nerve block; SCNB, supraclavicular nerve block; SSNB, suprascapular nerve block; HTESPB, high thoracic erector spinae plane block; CEB, cervical epidural block; ANB, axillary nerve block; IAI, intra-articular injection; OA, oral administration; EA, external application; IVA, intravenous administration; PL, placebo.

**Figure 8:**
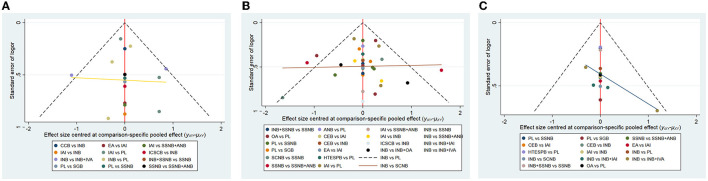
The comparison-adjusted funnel plot of network meta-analyses. [**(A)** postoperative 12 h, **(B)** postoperative 24 h, **(C)** postoperative 48 h]. INB, interscalene nerve block; SCNB, supraclavicular nerve block; SSNB, suprascapular nerve block; HTESPB, high thoracic erector spinae plane block; CEB, cervical epidural block; SGB, stellate ganglion block; ICSCB, infraclavicular-suprascapular blocks; CCB, costoclavicular blocks; ANB, axillary nerve block; IAI, intra-articular injection; OA, oral administration; EA, external application; IVA, intravenous administration; PL, placebo.

The publication bias of the 3 distinct networks was evaluated by using Egger's tests, and the result is shown in Table 10. The publication bias was only detected in network 2 (Table 6) due to the small amount of the subjects present in the studies included in this analysis. The rank possibility was recalculated by excluding these studies with <40 people. The results in postoperative 12 h changed significantly (Figures 7A–C). The small sample might produce bias, which can lead to the wrong ranking of ANB + SSNB (27). At present, it is considered that the larger sample size is more reliable for analysis. Moreover, in the network comparison, INB + SSNB was significantly better than ANB + SSNB, so it could be concluded that the analgesic effect of INB + SSNB ranked first in the 12 h group after the operation. A little asymmetry was found in the comparison–adjusted funnel plot, which suggested that there were small–study effects in the primary analysis (Figure 8).

**Table 10:** Egger's test for publication bias of pairwise meta–analysis.

**Group**	**Std_Eff**	**Coef**.	**Std. Err**.	**t**	**P>|t|**	**95% Conf. Interval**
Postoprative 12 h	Slope	−1.05	0.71	−1.49	0.16	(−2.56; 0.45)
	Bias	2.12	2.34	0.91	0.38	(−2.87; 7.11)
Postoprative 24 h	Slope	0.27	0.37	0.75	0.46	(−0.47; 1.02)
	Bias	−2.09	1.32	−1.58	0.12	(−4.8; 0.60)
Postoprative 48 h	Slope	0.05	0.48	0.10	0.93	(−0.99; 1.08)
	Bias	−1.90	1.65	−1.15	0.27	(−5.43; 1.64)

## Discussion

In this NMA, all the RCTs that focused on the different intervention measures in the treatment of pain after shoulder arthroscopy were included. The analgesic effects of the different interventions at postoperative 12 h, 24 h, and 48 h were analyzed, respectively. The intervention measures were divided into two distinct categories, namely, the nerve block group and the non–nerve block group. The results of SUCRA showed that, first, the analgesic effect of the nerve block group was significantly better than that of the non–nerve block group at the three time points after the operation. Among them, the first regimen related to the nerve was SSNB + INB at postoperative 12 h, INB + IAI at 24 h after operation, and INB + IAI at 48 h after surgery. For the non–nerve block group, the effect of EA was found to be the best in the 12 h after operation, and the analgesic effect of OA at postoperative 24 h and 48 h was significantly better than that of other intervention measures.

There was no intervention reported with INB + IAI and OA in the original data in the postoperative 12 h group (Figure 2), which might be the reason for the difference in results between 12 h and 24 h after operation. In addition, in the network comparison, the analgesic effect of OA at 24 h after operation was found to be significantly better than that of other intervention measures; however, SUCRA was ranked third in the 24 h group after the operation. The possible reasons could be related to the inadequate sample size of the experiments, the environment in which each experiment was carried out, and other external conditions, which might have exerted a variable impact on the experiment and so on.

### Clinical Implications

On the one hand, shoulder arthroscopic surgery is currently carried out successfully in a large number of affected patients. Thus, it can be implied that there are numerous patients undergoing shoulder arthroscopic surgery, and the postoperative pain can adversely slow down the recovery speed of the patients and affect the surgical effect on the patients. On the other hand, there is no unified and optimal scheme for postoperative analgesia after shoulder arthroscopic surgery. At present, the use of INB as the best nerve block has been recommended for postoperative pain after arthroscopic surgery (10), and it has been suggested to take analgesic drugs before and after shoulder arthroscopy. Moreover, IVA of dexamethasone can markedly increase the duration of anesthesia, reduce the use of anesthetic drugs, and alleviate the pain rebound after the disappearance of the anesthetic effect. Patients with pain can use opioid analgesics as per their requirements (3).

Dexamethasone or dexmedetomidine (54, 55), magnesium sulfate (56), or clonidine (57) can also be added to nerve block drugs, and intravenous anesthesia adjuvant drugs, such as ketamine (58), can also be injected into patients before and after the nerve block. A number of studies reported in the literature have been found to only block the upper trunk of the brachial plexus, which can achieve an effective analgesic effect equivalent to INB and can effectively reduce unilateral diaphragm paralysis (59). Moreover, the effect of continuous intermuscular sulcus nerve block has been found to be better than that of the single injection of intermuscular sulcus nerve block (60), and increasing drug concentration might effectively improve the anesthetic effect (61). Moreover, different types, concentrations, and volumes of local anesthetics may lead to significant clinical heterogeneity. Therefore, this point cannot be ignored in practical application.

Overall, the conclusion was drawn from this study that in the nerve block group, the analgesic effect of SSNB + INB was the best at postoperative 12 h, whereas INB + IAI was superior at postoperative 24 h and 48 h. For the non–nerve block group, the effect of EA was the best in the postoperative 12 h, and the analgesic effect of OA at postoperative 24 h and 48 h was significantly better as compared with other intervention measures.

In addition, in the non–nerve block group, patients can choose oral medicine before and after operation (11, 50), they can receive pain management education before operation (62), patients used an analgesic pump device after operation (13), and opioid analgesics were used after the operation, such as topical analgesic patch (19). Stellate ganglion block was not recommended because its analgesic effect was found to be significantly lower than that of other intervention strategies, and we recommended the application of a combination of multiple interventions to maximize the analgesic effect and reduce the side effects of a single drug.

However, this study does not include the various complications in the analysis, and the lowest incidences of complications in SSNB + ANB and INB + IAI intervention programs were unknown. In addition, it has been shown that injecting anesthetics into the articular cavity might damage the cartilage of patients and cause unexpected damage (63), so one should try to avoid injecting anesthetics directly on the surface of the cartilage and minimize the trauma.

### Implications for Future Research

According to the meta–analysis, the best analgesic effects were that of SSNB + INB, INB + IAI, and INB + IAI at the 3 time points after the operation, respectively. However, at postoperative 12 h, it was not clear whether the analgesic effect of SSNB + INB or INB + IAI was better, and hence clinical trials are needed to verify their efficacies in the future.

In addition, in the intervention control measures of each experiment, there were some other routine intervention measures used, which were not included in this NMA, such as the use of the postoperative analgesic pump, postoperative ice compress wound (64), and so on. Therefore, in addition to the above conclusions, we proposed that analgesics can be taken in advance before operation and use of nerve block such as INB plus IAI analgesics combined with postoperative analgesics, and cryotherapy in the ward, which may be the best analgesic intervention measures at the present.

In the future, high–quality RCTs should continue to be conducted to analyze the best multimode analgesic regimen for perioperative pain after shoulder arthroscopy.

### Limitations

This study is also associated with a few limitations. First, this article did not describe the possible side effects of each intervention, but we can conduct another relevant meta–analysis in the future to address this issue. Second, the lack of blind methods in some studies may lead to potential deviations in the effect. In addition, the risk that results may be influenced by the quality of the included RCTs of this article cannot be completely avoided, like any other meta–analysis. Moreover, the bias can also be introduced by the loss of patients during the follow–up, so it might be possible that the major complications were not properly reported. Finally, the inclusion of the various surgical methods and shoulder diseases in the literature is complex, and the meta–analysis of the surgical methods is not subdivided, which may cause potential bias. These can be further subdivided in the future when there are several other related clinical trials have been conducted.

## Conclusion

The analgesic effect of SSNB + INB was the best at postoperative 12 h, and INB + IAI was the best at postoperative 24 h and 48 h in the nerve block group. For the non–nerve block group, the effect of EA was the best at postoperative 12 h, and the analgesic effect of OA at postoperative 24 h and 48 h was significantly better than any other interventions.

## Data Availability Statement

The original contributions presented in the study are included in the article/supplementary material, further inquiries can be directed to the corresponding author/s.

## Author Contributions

WJ and QX collected data and wrote and revised the articles. LX, HS, YX, SG, NM, GT, DZ, SH, and XZ revised the articles. All authors contributed to the article and approved the submitted version.

## Funding

NM was supported by the Kuanren Talents Program of the Second Affiliated Hospital of Chongqing Medical University.

## Conflict of Interest

The authors declare that the research was conducted in the absence of any commercial or financial relationships that could be construed as a potential conflict of interest.

## Publisher's Note

All claims expressed in this article are solely those of the authors and do not necessarily represent those of their affiliated organizations, or those of the publisher, the editors and the reviewers. Any product that may be evaluated in this article, or claim that may be made by its manufacturer, is not guaranteed or endorsed by the publisher.

## Abbreviations

INB: interscalene nerve block
SCNB: supraclavicular nerve block
SSNB: suprascapular nerve block
HTESPB: high thoracic erector spine plane block
CEB: cervical epidural block
SGB: stellate ganglion block
ICSCB: infraclavicular-suprascapular block
CCB: costoclavicular block
ANB: axillary nerve block
IAI: intra-articular injection
PL: placebo
OA: oral administration
EA: external application
IVA: intravenous administration
SUCRA: surface under the cumulative ranking curve
VAS: visual analog scale
NRS: numeric rating scale
M ± SD: = mean ± standard deviation
MD and 95%CI: mean difference and 95% confidence interval
SMD: standard mean difference
RCT: randomized controlled trial
NMA: network meta-analysis.
